# Bridging the Gap in Antipsychotic Safety: A Translational Audit of Physical Health Monitoring in an Assertive Outreach Team

**DOI:** 10.1192/bjo.2026.11838

**Published:** 2026-06-30

**Authors:** Jasbinderbir Singh, Gurjot Bhangoo, Hanna Leech, Salim AL-Husieni

**Affiliations:** 1 College of Medicine and Health Science, University of Birmingham, Birmingham, United Kingdom; 2 Birmingham and Solihull Mental Health NHS Foundation Trust, Birmingham, United Kingdom

## Abstract

**Aims:**

Individuals with severe and enduring mental illness face a significantly elevated risk of developing cardiometabolic diseases. The side effects of antipsychotic medications–such as weight gain, diabetes, and QTc prolongation–can exacerbate these risks. Consequently, NICE guidelines recommend annual monitoring of physical health parameters for all patients on antipsychotic medication. This audit aimed to evaluate the compliance of monitoring practices within an Assertive Outreach Team with these recommendations and identify gaps between guidelines and clinical practice.

**Methods:**

A retrospective clinical audit was conducted involving patients managed by a Birmingham Assertive Outreach Team who had received antipsychotic treatment for at least 12 months between December 2023 and December 2024. Electronic health records were reviewed for documentation of annual measurements, including BMI, blood pressure, ECG, and blood investigations (FBC, LFTs, lipids, HbA1c, and U&Es). This data was stored electronically and analysed using Excel. Compliance was assessed against the NICE standard of 100% for the annual completion of each health test.

**Results:**

Seventy-four patients met the inclusion criteria. Only 18% received all recommended monitoring within the review period. Among non-compliant cases, 41% were missing three or more investigations, with BMI and ECGs being the most frequently omitted cardiometabolic measures.

**Conclusion:**

Monitoring of physical health in this high-risk patient population fell significantly short of the NICE standards. Implementing simple system-level interventions, such as structured consultation checklists, electronic reminders, and regular staff education, may enhance adherence to guidelines. A re-audit is planned to assess the impact of these recommendations and support sustainable quality improvement across community mental health services.

